# Clinical phenotypes and outcomes associated with SARS-CoV-2 Omicron sublineage JN.1 in critically ill COVID-19 patients: a prospective, multicenter cohort study in France, November 2022 to January 2024

**DOI:** 10.1186/s13613-024-01319-w

**Published:** 2024-06-28

**Authors:** Nicolas de Prost, Etienne Audureau, Antoine Guillon, Lynda Handala, Sébastien Préau, Aurélie Guigon, Fabrice Uhel, Quentin Le Hingrat, Flora Delamaire, Claire Grolhier, Fabienne Tamion, Alice Moisan, Cédric Darreau, Jean Thomin, Damien Contou, Amandine Henry, Thomas Daix, Sébastien Hantz, Clément Saccheri, Valérie Giordanengo, Tài Pham, Amal Chaghouri, Pierre Bay, Jean-Michel Pawlotsky, Slim Fourati, the SEVARVIR investigators, Keyvan Razazi, Armand Mekontso Dessap, Raphaël Bellaïche, Lucile Picard, Alexandre Soulier, Mélissa N’Debi, Sarah Seng, Christophe Rodriguez, Frédéric Pene, Anne-Sophie L’Honneur, Adrien Joseph, Elie Azoulay, Maud Salmona, Marie-Laure Chaix, Charles-Edouard Luyt, David Levy, Julien Mayaux, Stéphane Marot, Juliette Bernier, Maxime Gasperment, Tomas Urbina, Hafid Ait-Oufella, Eric Maury, Laurence Morand-Joubert, Djeneba Bocar Fofana, Jean-François Timsit, Diane Descamps, Guillaume Voiriot, Nina de Montmollin, Mathieu Turpin, Stéphane Gaudry, Ségolène Brichler, Tài Olivier Pham, Elyanne Gault, Sébastien Jochmans, Aurélia Pitsch, Guillaume Chevrel, Céline Clergue, Kubab Sabah, Laurence Courdavault Vagh Weinmann, Claudio Garcia-Sanchez, Ferhat Meziani, Louis-Marie Jandeaux, Samira Fafi-Kremer, Elodie Laugel, Sébastien Preau, Aurélie Guignon, Antoine Kimmoun, Evelyne Schvoerer, Cédric Hartard, Charles Damoisel, Nicolas Brechot, Helene Péré, François Beloncle, Francoise Lunel Fabiani, Rémi Coudroy, Arnaud W Thille, François Arrive, Sylvain le Pape, Laura Marchasson, Luc Deroche, Nicolas Leveque, Vincent Thibaut, Béatrice la Combe, Séverine Haouisee, Alexandre Boyer, Sonia Burrel, Gaetan Beduneau, Christophe Girault, Maximillien Grall, Dorothée Carpentier, Jean-Christophe Plantier, Emmanuel Canet, Audrey Rodallec, Berthe Marie Imbert, Sami Hraeich, Pierre-Edouard Fournier, Philippe Colson, Anaïs Dartevel, Sylvie Larrat, Guillaume Thiery, Sylvie Pillet, Kada Klouche, Edouard Tuaillon, Cécile Aubron, Adissa Tran, Sophie Vallet, Pierre-Emmanuel Charles, Alexis le Rougemont, Bertrand Souweine, Cecile Henquell, Audrey Mirand, Bruno Mourvillier, Laurent Andreoletti, Clément Lier, Damien du Cheyron, Nefert Candace Dossou, Astrid Vabret, Gaël Piton, Quentin Lepiller, Sylvie Roger

**Affiliations:** 1 Médecine Intensive Réanimation, Hôpitaux Universitaires Henri Mondor, Assistance Publique – Hôpitaux de Paris (AP-HP), Créteil, France; 2 Groupe de Recherche Clinique CARMAS, Université Paris-Est-Créteil (UPEC), Créteil, France; 3 Université Paris-Est-Créteil (UPEC), Créteil, France; 4 INSERM U955, Team Viruses, Hepatology, Cancer, Créteil, France; 5 Department of Public Health, Hôpitaux Universitaires Henri Mondor, Assistance Publique – Hôpitaux de Paris (AP-HP), Créteil, France; 6 IMRB INSERM U955, Team CEpiA, Créteil, France; 7 Intensive Care Unit, Research Center for Respiratory Diseases (CEPR), INSERM U1100, Tours University Hospital, University of Tours, Tours, France; 8 INSERM U1259, Université de Tours, Tours, France; 9 U1167 – RID-AGE Facteurs de Risque et Déterminants Moléculaires des Maladies Liées au Vieillissement, University Lille, Inserm, CHU Lille, Institut Pasteur de Lille, 59000 Lille, France; 10 Service de Virologie, CHU de Lille, 59000 Lille, France; 11 DMU ESPRIT, Service de Médecine Intensive Réanimation, Université Paris Cité, APHP, Hôpital Louis Mourier, Colombes, France; 12 INSERM UMR-S1151, CNRS UMR-S8253, Institut Necker-Enfants Malades (INEM), Université Paris Cité, Paris, France; 13 IAME INSERM UMR 1137, Service de Virologie, Université Paris Cité, Hôpital Bichat-Claude Bernard, Assistance Publique – Hôpitaux de Paris, Paris, France; 14 CHU Rennes, Maladies Infectieuses et Réanimation Médicale, Rennes, France; 15 Laboratoire de Virologie, CHU Rennes, Rennes, France; 16 Service de Médecine Intensive-Réanimation, CHU De Rouen, 76000 Rouen, France; 17 INSERM DYNAMICURE UMR 1311 Department of Virology, Univ Rouen Normandie, Université de Caen Normandie, Normandie Univ, CHU Rouen, National Reference Center of HIV, 76000 Rouen, France; 18 Service de Réanimation Médico-Chirurgicale, Centre Hospitalier du Mans, Le Mans, France; 19 Laboratoire de Microbiologie, Centre Hospitalier du Mans, Le Mans, France; 20 Service de Réanimation, Hôpital Victor Dupouy, Argenteuil, France; 21 Service de Virologie, Hôpital Victor Dupouy, Argenteuil, France; 22 INSERM CIC 1435 and UMR 1092, Réanimation Polyvalente, CHU Limoges, Limoges, France; 23 Bacteriology, Virology, Hygiene Department, French National Reference Center for Herpesviruses, CHU Limoges, 87000 Limoges, France; 24 Service de Réanimation Médicale, CHU de Nice, Nice, France; 25 Laboratoire de Virologie, CHU de Nice, Nice, France; 26 Service de Médecine Intensive-Réanimation, DMU 4 CORREVE Maladies du Cœur Et Des Vaisseaux, Assistance Publique – Hôpitaux de Paris, Hôpital de Bicêtre, FHU Sepsis, Le Kremlin-Bicêtre, France; 27 Inserm U1018, Equipe d’Epidémiologie Respiratoire Intégrative, CESP, 94807 Villejuif, France; 28 Laboratoire de Virologie, Hôpital Paul Brousse, Assistance Publique – Hôpitaux de Paris, Villejuif, France; 29 Department of Virology, Hôpitaux Universitaires Henri Mondor, Assistance Publique – Hôpitaux de Paris, Créteil, France; 30 National Reference Center for HIV-Associated Laboratory, , CHRU de Tours, Tours, France; 31 INSERM, RESINFIT, U1092, 87000 Limoges, France; 32 Service de Médecine Intensive Réanimation, Hôpital Henri Mondor, Créteil, France

**Keywords:** SARS-CoV-2, Omicron, Subvariant, JN.1, Acute respiratory failure

## Abstract

**Background:**

A notable increase in severe cases of COVID-19, with significant hospitalizations due to the emergence and spread of JN.1 was observed worldwide in late 2023 and early 2024. However, no clinical data are available regarding critically-ill JN.1 COVID-19 infected patients.

**Methods:**

The current study is a substudy of the SEVARVIR prospective multicenter observational cohort study. Patients admitted to any of the 40 participating ICUs between November 17, 2022, and January 22, 2024, were eligible for inclusion in the SEVARVIR cohort study (NCT05162508) if they met the following inclusion criteria: age ≥ 18 years, SARS-CoV-2 infection confirmed by a positive reverse transcriptase-polymerase chain reaction (RT-PCR) in nasopharyngeal swab samples, ICU admission for acute respiratory failure. The primary clinical endpoint of the study was day-28 mortality. Evaluation of the association between day-28 mortality and sublineage group was conducted by performing an exploratory multivariable logistic regression model, after systematically adjusting for predefined prognostic factors previously shown to be important confounders (i.e. obesity, immunosuppression, age and SOFA score) computing odds ratios (OR) along with their corresponding 95% confidence intervals (95% CI).

**Results:**

During the study period (November 2022–January 2024) 56 JN.1- and 126 XBB-infected patients were prospectively enrolled in 40 French intensive care units. JN.1-infected patients were more likely to be obese (35.7% vs 20.8%; p = 0.033) and less frequently immunosuppressed than others (20.4% vs 41.4%; p = 0.010). JN.1-infected patients required invasive mechanical ventilation support in 29.1%, 87.5% of them received dexamethasone, 14.5% tocilizumab and none received monoclonal antibodies. Only one JN-1 infected patient (1.8%) required extracorporeal membrane oxygenation support during ICU stay (vs 0/126 in the XBB group; p = 0.30). Day-28 mortality of JN.1-infected patients was 14.6%, not significantly different from that of XBB-infected patients (22.0%; p = 0.28). In univariable logistic regression analysis and in multivariable analysis adjusting for confounders defined a priori, we found no statistically significant association between JN.1 infection and day-28 mortality (adjusted OR 1.06 95% CI (0.17;1.42); p = 0.19). There was no significant between group difference regarding duration of stay in the ICU (6.0 [3.5;11.0] vs 7.0 [4.0;14.0] days; p = 0.21).

**Conclusions:**

Critically-ill patients with Omicron JN.1 infection showed a different clinical phenotype than patients infected with the earlier XBB sublineage, including more frequent obesity and less immunosuppression. Compared with XBB, JN.1 infection was not associated with higher day-28 mortality.

**Supplementary Information:**

The online version contains supplementary material available at 10.1186/s13613-024-01319-w.

## Background

Following the emergence of the Omicron variant of SARS-CoV-2, several sublineages have co-circulated until the dominance of XBB recombinant variants in early 2023, which were subsequently replaced by a distinct branch of BA.2 named BA.2.86. Compared to XBB and the parental BA.2, the spike protein of BA.2.86 has more than 30 mutations [1]. Initially, BA.2.86 did not dominate other coexisting subvariants until it acquired an additional mutation (i.e., L455S), causing its progeny JN.1 to rapidly increase and become the dominant SARS-CoV-2 sublineage in several parts of the world. Subsequently, the WHO designated JN.1 as a variant of interest due to its increased transmissibility.

Several in vitro studies have shown that JN.1 has phenotypic characteristics that confer enhanced in vitro fitness. The L455S substitution in the spike protein enhances the ability of the virus to bind to the angiotensin-converting enzyme 2 receptor. JN.1 also appears to be one of the most immune-evading SARS-CoV-2 variants to date, contributing to its increased transmissibility compared to other Omicron sublineages [2].

Clinical reports from medical institutions indicate that the risk of serious illness due to JN.1 variant infection is low [3]. However, there has been a notable increase in severe cases of COVID-19, with significant hospitalizations due to COVID-19 in late 2023. Importantly, a certain proportion of patients is still admitted to intensive care units (ICUs) for COVID-19-associated acute respiratory failure, but their clinical phenotype and outcomes have changed since the early waves of the pandemic [4, 5], and those of patients admitted with severe COVID-19 due to the JN.1 subvariant are currently unknown. This information is critical as it could improve our ability to target individuals who may benefit from more personalized preventive measures, such as frequent vaccination and/or active immunoprophylaxis, as well as tailored therapeutic interventions, including early administration of antivirals in the event of infection.

As part of the SEVARVIR study, we have established a prospective French national multicenter cohort focused on patients admitted to ICUs with COVID-19-associated acute respiratory failure. In this specific substudy, our aims are (1) to assess day-28 mortality and (2) to comprehensively characterize the clinical presentation of patients infected with the emerging JN.1 variant and compare them with those infected with sublineages derived from XBB.

## Methods

### Study design and patients

The current study is a substudy of the SEVARVIR prospective multicenter observational cohort study. Patients admitted to any of the 40 participating ICUs between November 17, 2022, and January 22, 2024, were eligible for inclusion in the SEVARVIR cohort study (NCT05162508, see Supplementary Table 1 for the list of participating centers) if they met the following inclusion criteria: age ≥ 18 years, SARS-CoV-2 infection confirmed by a positive reverse transcriptase-polymerase chain reaction (RT-PCR) in nasopharyngeal swab samples, ICU admission for acute respiratory failure (i.e., peripheral oxygen saturation ≤ 90% and need for supplemental oxygen or any type of ventilatory support). Patients with SARS-CoV-2 infection but no acute respiratory failure or with a RT-PCR cycle threshold (Ct) value > 32 in nasopharyngeal swabs were not included. The study was approved by the Comité de Protection des Personnes Sud-Méditerranée I (N° EudraCT/ID-RCB: 2021-A02914-37). Informed consent was obtained from all patients or their relatives.

Demographics, clinical and laboratory variables were recorded upon ICU admission and during ICU stay. Patients’ frailty was assessed using the Clinical Frailty Scale [6]. The severity of the disease upon ICU admission was assessed using the World Health Organization (WHO) 10-point ordinal scale [7], the sequential organ failure assessment (SOFA [8]) score, and the simplified acute physiology score (SAPS [9]) II score. Acute respiratory distress syndrome (ARDS) was defined according to the Berlin definition [10]. Immunosuppression was defined as solid-organ transplant, active onco-hematological malignancy (within the past three years), HIV infection, long-term corticosteroid treatment (i.e., more than three months of > 0.5 mg/kg/day prednisone equivalent), and exposure to any other immunosuppressive treatment. Obesity was defined as a body mass index greater than 30 kg/m^2^. The primary clinical endpoint of the study was day-28 mortality.

### SARS-CoV-2 variant determination

Full-length SARS-CoV-2 genomes from all included patients were sequenced by means of next-generation sequencing. For mutational pattern analysis at the amino acid level, only high-quality sequences, i.e., sequences covering ≥ 90% of the viral genome and 95% of the spike gene, were considered. Full-length viral genome sequence analysis yielding high coverage will be deposited in Genbank.

### Statistical analysis

Descriptive results are presented as mean ± standard deviation [SD] or median (1st-3rd quartiles) for continuous variables, and as numbers with percentages for categorical variables. Exploratory unadjusted comparisons between patients infected with two groups of Omicron sublineages (including XBB sublineages, referred to as the “XBB group”, and emerging BA.2.86 sublineages, [parental BA.2.86, JN.1, and JN.3], referred to as the “JN.1 group”) were performed using Chi-squared or Fisher’s exact tests for categorical variables, and ANOVA or Kruskal–Wallis tests for continuous variables, as appropriate. Evaluation of the association between day-28 mortality and sublineage group was conducted by performing an exploratory multivariable logistic regression model, after systematically adjusting for predefined prognostic factors previously shown to be important confounders, i.e. body mass index, immunosuppression, age and SOFA score, computing odds ratios (OR) along with their corresponding 95% confidence intervals (95% CI).

The overall sample size of the SEVARVIR study was a priori defined (n = 2000). The sample size of this substudy was not predefined. Indeed, we had anticipated that data could be sequentially extracted from the prospective database based on epidemiological surges. Results have been reported according to the STROBE guidelines for cohort studies (Supplementary Table 2).

Two-sided p-values < 0.05 were considered statistically significant. No missing data imputation was performed and analyses were performed on complete cases. Analyses were performed with Stata V16.1 statistical software (StataCorp, College Station, TX, USA) and R 4.2.0 (R Foundation for Statistical Computing, Vienna, Austria).

## Results

**Fig. 1:**
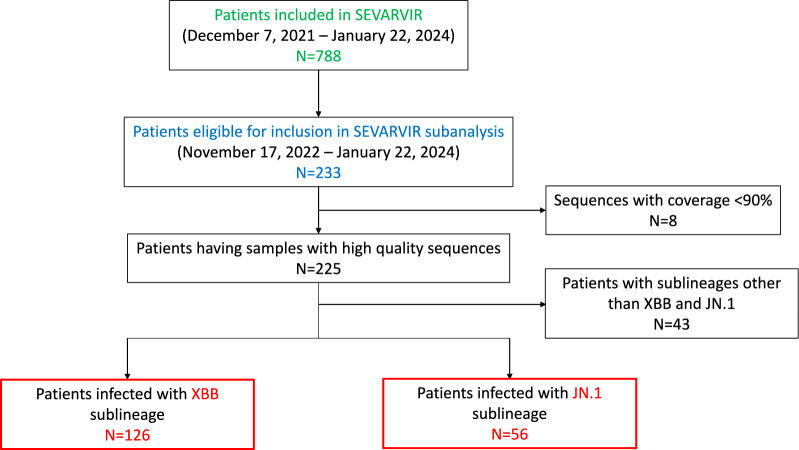
Study flow chart. The flow chart depicts the total number of patients included in the SEVARVIR cohort study since the inclusion of the first patient (December, 7 2021; n = 788) and during the XBB and JN.1 epidemic waves (n = 233)

Between November 17, 2022, and January 22, 2024, 233 patients were admitted to one of the 40 participating ICUs and enrolled in the SEVARVIR cohort study. Of these, 126 patients in the “XBB group” and 56 patients in the “JN.1 group” were included in the analysis (Fig. 1).

### The clinical phenotype of JN.1-infected patients differed from that of XBB-infected patients

No statistically significant differences were observed between patients infected with JN.1 and XBB sublineages with respect to age, gender and frequency of comorbidities. However, patients in the JN.1group were more likely to be obese (n = 20/56, 35.7% vs n = 26/125, 20.8%; p = 0.033), and had a statistically significant higher median body mass index (26.4 [22.4–33.4] vs 25.0 [21.2–28.7] kg/m^2^; p = 0.019). There were also significantly fewer immunosuppressed patients in the JN.1 group than in the XBB group (n = 10/49, 20.4% vs n = 48/116, 41.4%; p = 0.010) (Table 1).

**Table 1:** Clinical and biological characteristics of the 182 patients with severe SARS-CoV-2 infection at the time of their intensive care unit admission according to the infecting SARS-CoV-2 “sublineage groups” (XBB *vs* JN.1 group)

		Data available	All patients	XBB group	JN.1 group	p-value
			N = 182	N = 126	N = 56	
Demographics and comorbidities						
Sex, females		182	67 (36.8%)	50 (39.7%)	17 (30.4%)	0.23
Age, years		182	70.7 [62.9;76.2]	71.1 [63.2;75.9]	69.5 [62.4;76.8]	0.74
Diabetes		165	61 (37.0%)	41 (35.3%)	20 (40.8%)	0.51
Obesity		181	46 (25.4%)	26 (20.8%)	20 (35.7%)	**0.033**
Body mass index, kg/m^2^		181	25.5 [21.8;30.1]	25.0 [21.2;28.7]	26.4 [22.5;33.2]	**0.035**
Chronic heart failure		164	27 (16.5%)	16 (13.9%)	11 (22.4%)	0.18
Hypertension		165	96 (58.2%)	65 (56.0%)	31 (63.3%)	0.39
Chronic respiratory failure^a^		165	45 (27.3%)	31 (26.7%)	14 (28.6%)	0.81
Chronic renal failure^b^		165	35 (21.2%)	25 (21.6%)	10 (20.4%)	0.87
Cirrhosis		165	3 (1.8%)	2 (1.7%)	1 (2.0%)	> 0.99
Immunosuppression		165	58 (35.2%)	48 (41.4%)	10 (20.4%)	**0.010**
Immunosuppression	None	164	107 (65.2%)	68 (58.6%)	39 (81.3%)	0.059
	Solid organ transplant		11 (6.7%)	9 (7.8%)	2 (4.2%)	
	Onco-hematological malignancies		27 (16.5%)	23 (19.8%)	4 (8.3%)	
	Others^c^		19 (11.6%)	16 (13.8%)	3 (6.3%)	
Number of comorbidities^d^		166	2.00 [1.00;3.00]	2.00 [1.00;3.00]	2.00 [1.00;3.00]	0.46
Clinical frailty scale		179	3.00 [2.00;4.00]	3.00 [2.00;5.0]	3.00 [2.00;4.00]	0.59
SARS-CoV-2 infection and vaccination						
Previous SARS-CoV-2 infection		145	26 (17.9%)	19 (19.4%)	7 (14.9%)	0.51
SARS-CoV-2 vaccination		148	123 (83.1%)	83 (79.8%)	40 (90.9%)	0.10
Number of doses among vaccinated		111	3.00 [3.00;4.00]	3.00 [3.00;4.00]	3.00 [3.00;3.00]	**0.020**
Last dose—ICU admission^e^, days		41	554 [265;680]	315 [189;631]	628 [375;691]	0.067
SARS-CoV-2 serology at ICU admission	Unavailable	182	143 (78.6%)	96 (76.2%)	47 (83.9%)	0.52
	Negative^f^		7 (3.8%)	6 (4.8%)	1 (1.8%)	
	Positive		32 (17.6%)	24 (19.0%)	8 (14.3%)	
First symptoms—ICU admission, days		182	5.0 [2.00;8.0]	5.0 [3.00;9.0]	3.00 [1.00;6.0]	**0.006**
SARS-CoV-2 RNA detection in nasopharyngeal swabs, Ct		142	19.5 [16.0;22.8]	19.0 [16.0;22.0]	20.0 [17.0;23.0]	0.65
Patients severity upon ICU admission and biological features						
WHO 10-point scale		182	6 (6;6)	6 (6;6)	6 (6;7)	0.329
SAPS II score		153	39.0 [30.0;49.0]	39.0 [31.0;49.5]	37.0 [26.3;48.5]	0.37
SOFA score		163	4.00 [3.00;7.0]	4.00 [3.00;7.0]	4.00 [3.50;7.0]	0.42
PaO_2_/FiO_2_ ratio, mmHg		177	138 [92.9;197]	131 [93.0;193]	146 [92.0;211]	0.60
ARDS criteria		177	137 (77.4%)	94 (77.0%)	43 (78.2%)	0.87
Arterial lactate level, mM		161	1.50 [1.10;2.20]	1.55 [1.10;2.20]	1.50 [1.10;2.20]	0.98
Blood leukocytes, G/L		176	9.3 [5.9;14.7]	8.9 [4.80;13.4]	10.7 [7.6;17.0]	**0.019**
Blood lymphocytes, G/L		152	0.50 [0.30;1.10]	0.50 [0.30;0.80]	0.60 [0.40;1.60]	0.052
Blood platelets, G/L		175	204 [139;276]	195 [129;280]	212 [165;273]	0.24
Serum urea level, mM		174	9.0 [6.0;15.0]	9.0 [6.0;14.0]	9.0 [5.8;17.0]	0.87
Serum creatinine level, µM		178	95.5 [65.0;158]	93.5 [61.8;159]	97.0 [69.0;153]	0.47
Bacterial coinfection		181	49 (27.1%)	29 (23.0%)	20 (36.4%)	0.063
Thoracic CT-scan						
Pulmonary embolism		181	10 (5.5%)	5 (4.0%)	5 (8.9%)	0.29
Lung parenchyma involvement, %		60	50.0 [25.0;66.3]	45.0 [25.0;64.3]	50.0 [26.3;68.8]	0.42
Oxygen/ventilatory support	Oxygen	182	33 (18.1%)	24 (19.0%)	9 (16.1%)	0.68
	High flow oxygen		76 (41.8%)	55 (43.7%)	21 (37.5%)	
	NIV/C-PAP		33 (18.1%)	22 (17.5%)	11 (19.6%)	
	Invasive MV		40 (22.0%)	25 (19.8%)	15 (26.8%)	
ECMO		182	0 (0.0%)	0 (0.0%)	0 (0.0%)	–
Vasopressor support		177	30 (16.9%)	20 (16.4%)	10 (18.2%)	0.77

Results are N(%), means (± standard deviation) or medians (interquartile range)

ARDS: acute respiratory distress syndrome; ICU: intensive care unit; Ct: cycle threshold; WHO: World Health Organization; SOFA: Sequential Organ Failure Assessment; SAPS II: Simplified Acute Physiology Score II; NIV: non-invasive ventilation; C-PAP; continuous-positive airway pressure; MV: mechanical ventilation; ECMO: extracorporeal mechanical ventilation; Two-tailed p-values come from unadjusted comparisons using Chi square or Fisher’s exact tests for categorical variables, and t-tests or Mann–Whitney tests for continuous variables, as appropriate. No adjustment for multiple comparisons was performed; Bolded p-values are significant at the p < 0.05 level

^a^requiring long-term oxygen treatment; ^b^defined as glomerular filtration rate < 60 mL/min/1,73m^2 c^includes HIV infection, long-term corticosteroid treatment, and other immunosuppressive treatments; ^d^include diabetes, obesity, chronic heart, renal and respiratory failure, hypertension, cirrhosis, and immunosuppression; ^e^time lag between the last vaccination dose and ICU admission; ^f^defined as < 30 Binding Antibody Units (BAU)/mL

The proportion of patients who had received at least one dose of SARS-CoV-2 vaccine did not show significant statistical differences between groups, although the median number of doses received was significantly higher in patients from the XBB group (3 [3–4] vs 3 [3–3]; p = 0.019). The median time from onset of first symptoms to ICU admission was significantly shorter in the JN.1 group than in the XBB group (3 [1–6] vs 5 [3–9] days; p = 0.006). Other variables related to SARS-CoV-2 virological characteristics, including median viral level in the upper respiratory tract measured by cycle threshold in RT-PCR and prevalence of positive SARS-CoV-2 anti-S antibodies at ICU admission, did not differ statistically significantly between groups (Table 1).

There was no statistically significant difference between the two groups in the severity of illness at ICU admission, as reflected by the SOFA and SAPS II scores and the WHO 10-point ordinal scale (Table 1). Invasive mechanical ventilation support was required in 22.0% (n = 40/182) of patients within 24 h of ICU admission, with no statistically significant difference between groups. No patient required extracorporeal membrane oxygenation (ECMO) support on ICU admission. Respiratory failure was eventually attributed to SARS-CoV-2 pneumonia without bacterial co-infection in about half of cases in both JN.1 and XBB groups (Table 2).

**Table 2:** Characteristics and mechanisms of acute respiratory failure in patients with severe SARS-CoV-2 infection (n = 182) according to the SARS-CoV-2 infecting “sublineage groups” (XBB *vs* JN.1 group)

	Data available	All patients	XBB group	JN.1 group	p-value
		N = 182	N = 126	N = 56	
Documented bacterial coinfection	181	49 (27.1%)	29 (23.0%)	20 (36.4%)	0.063
Thoracic CT scan					
Pulmonary embolism	181	10 (5.5%)	5 (4.0%)	5 (8.9%)	0.29
Lung parenchyma involvement, %	60	50.0 [25.0;66.3]	45.0 [25.0;64.3]	50.0 [26.3;68.8]	0.42
Final diagnosis of acute respiratory failure^a^	167				
SARS-CoV-2 pneumonia without bacterial co-infection		83 (49.7)	59 (52.7)	24 (43.6)	0.588
SARS-CoV-2 pneumonia with bacterial superinfection		56 (33.5)	33 (29.5)	23 (41.8)	
Cardiogenic pulmonary edema		5 (3.0)	4 (3.6)	1 (1.8)	
Decompensated chronic respiratory failure		11 (6.6)	8 (7.1)	3 (5.5)	
Other		12 (7.2)	8 (7.1)	4 (7.3)	

Results are N (%), means (± standard deviation) or medians (interquartile range)

MV: mechanical ventilation; ECMO: extracorporeal mechanical ventilation; VAP: ventilator-acquired pneumonia; IMV: invasive mechanical ventilation; CAPA: COVID-19-associated pulmonary aspergillosis

^a^ Retained by clinicians at the end of intensive care unit stay; Two-tailed p-values come from unadjusted comparisons using Chi square or Fisher’s exact tests for categorical variables, and t-tests or Mann–Whitney tests for continuous variables, as appropriate. No adjustment for multiple comparisons was performed; **Bolded** p-values are significant at the p < 0.05 level

### Day-28 mortality in JN.1-infected patients did not differ from that of XBB-infected patients

Day-28 mortality was not statistically significantly different between JN.1- and XBB-infected patients (14.6%, n = 7/48 vs 22.0%, n = 27/124; p = 0.28) (Table 3). In univariable logistic regression analysis and in multivariable analysis adjusting for confounders defined a priori, we found no statistically significant association between the infecting sublineage and day-28 mortality (Table 4). Age and a body mass index < 18 kg/cm^2^ showed a statistically significant association with day-28 mortality.

**Table 3:** Intensive care management and outcomes of patients with severe SARS-CoV-2 infection (n = 182) during their intensive care unit stay according to the SARS-CoV-2 infecting “sublineage groups” (XBB *vs* JN.1 group)

	Data available	All patients	XBB group	JN.1 group	p-value
		N = 182	N = 126	N = 56	
Invasive MV	181	59 (32.6%)	43 (34.1%)	16 (29.1%)	0.51
Prone positioning	170	32 (18.8%)	26 (21.7%)	6 (12.0%)	0.14
MV duration, days	56	8.0 [3.75;12.3]	8.0 [4.0;14.0]	4.0 [3.0;9.0]	0.16
Live-ventilator free days at day 28	173	28.0 [15.0;28.0]	28.0 [0.0;28.0]	28.0 [23.3;28.0]	0.22
ECMO support	181	1 (0.6%)	0 (0.0%)	1 (1.8%)	0.30
Vasopressor support	181	49 (27.1%)	38 (30.2%)	11 (20.0%)	0.16
Renal replacement therapy	181	22 (12.2%)	17 (13.5%)	5 (9.1%)	0.40
Ventilator-acquired pneumonia (among IMV)^a^	59	19 (32.2%)	17 (39.5%)	2 (12.5%)	0.048
CAPA	181	6 (3.3%)	6 (4.8%)	0 (0.0%)	0.18
Dexamethasone	159	108 (67.9%)	66 (59.5%)	42 (87.5%)	** < 0.001**
Tocilizumab	181	22 (12.2%)	14 (11.1%)	8 (14.5%)	0.52
Nirmatrelvir-ritonavir	181	4 (2.2%)	3 (2.4%)	1 (1.8%)	> 0.99
Monoclonal antibodies	180	1 (0.6%)	1 (0.8%)	0 (0.0%)	> 0.99
Duration of ICU stay, days	158				
All patients	180	6.0 [4.0;13.0]	7.0 [4.0;14.0]	6.0 [3.5;11.0]	0.21
In survivors at day 28 only	136	6.0 [4.0;13.3]	6.0 [4.0;14.0]	6.0 [4.0;11.0]	0.52
Day-28 mortality	171	34 (19.9%)	27 (22.0%)	7 (14.6%)	0.28

Results are N (%), means (± standard deviation) or medians (interquartile range)

MV: mechanical ventilation; ECMO: extracorporeal mechanical ventilation; VAP: ventilator-acquired pneumonia; IMV: invasive mechanical ventilation; CAPA: COVID-19-associated pulmonary aspergillosis

^a^ VAP episodes were recorded per definition in patients under IMV since more than 48 h; Two-tailed p-values come from unadjusted comparisons using Chi square or Fisher’s exact tests for categorical variables, and t-tests or Mann–Whitney tests for continuous variables, as appropriate. No adjustment for multiple comparisons was performed; **Bolded** p-values are significant at the p < 0.05 level

**Table 4:** Association between SARS-CoV-2 infecting “sublineage groups” (XBB *vs* JN.1 group) and day-28 mortality: results from univariable and multivariable logistic regression modeling adjusting for predefined prognostic factors

	Descriptive statistics			Univariable analysis			Multivariable analysis		
Characteristic	Alive N = 137^1^	Dead N = 34^1^	p-value^2^	N	OR (95% CI)^3^	p-value	N	aOR (95% CI)^4^	p-value
Sublineage group			0.28	171		0.28	140		0.20
XBB	96 (70.1%)	27 (79.4%)			—			—	
JN.1	41 (29.9%)	7 (20.6%)			0.61 (0.24; 1.51)	0.28		0.49 (0.17; 1.45)	0.20
Age, years	70.2 [60.7;75.7]	73.4 [69.2;76.8]	**0.023**	171	1.06 (1.01; 1.11)	**0.012**	140	1.08 (1.02; 1.14)	**0.011**
SOFA score	4.00 [3.00;7.0]	6.0 [3.25;7.0]	0.074	153	1.10 (0.97; 1.24)	0.14	140	1.12 (0.98; 1.28)	0.10
Body mass index, categorical			0.41	170		0.45	140		0.075
< 18 kg/cm^2^	6 (4.4%)	3 (8.8%)			2.31 (0.53; 9.98)	0.26		6.99 (1.14; 42.81)	**0.036**
[18–30] kg/cm^2^	97 (71.3%)	21 (61.8%)			—			—	
≥ 30 kg/cm^2^	33 (24.3%)	10 (29.4%)			1.40 (0.60; 3.28)	0.44		1.97 (0.71; 5.50)	0.19
Immunosuppression	44 (34.9%)	12 (37.5%)	0.79	158	1.12 (0.50; 2.50)	0.79	140	1.45 (0.55; 3.83)	0.45

^1^n (%); Median [25%; 75%]

^2^Pearson's Chi-squared test; Wilcoxon rank sum test; Fisher's exact test

^3^Odds ratio (95% confidence interval) from unadjusted logistic regression modeling

^4^adjusted odds ratio (95% confidence interval) from multivariable logistic regression modeling

Bolded p-values are significant at the p < 0.05 level

During the ICU stay, 32.6% (n = 59/181) of patients required invasive mechanical ventilation, with no statistical significant differences between the subgroups. There was also no statistical significant difference between groups regarding the need for other organ support (Table 3). Regarding COVID-19 management, JN.1-infected patients were treated with dexamethasone significantly more often than their XBB counterparts (n = 42/48, 87.5% vs n = 66/111, 59.5%; p < 0.001). No statistically significant differences between groups were observed in the use of other treatments, including anti-IL-6 antagonists, convalescent plasma and antivirals (Table 3).

## Discussion

The current study is the first to describe the in-hospital mortality and the clinical phenotype associated with the newly emerging Omicron sublineage JN.1 in patients with severe COVID-19 requiring ICU admission. Our data provide reassuring evidence that this emerging sublineage does not cause more severe outcomes than XBB variants that emerged and spread earlier in the population. We observed unexpected phenotypic differences, with more frequent obesity and less frequent immunosuppression in patients infected with JN.1, as compared to those infected with XBB sublineages.

The main result of our study is that day-28 mortality of JN.1-infected patients did not significantly differ from that of XBB-infected patients. Recent epidemiologic data have confirmed the increased transmissibility of JN.1. Its proportion of the circulating variants in the US had increased to more than 90% according to nowcast estimates from the US Centers for Disease Control and Prevention (CDC) [11]. In France, JN.1 represented more than 90% of circulating variants, according to the *Santé Publique France* report of January 31st, 2024 [12]. In this context, and given the surge in COVID-19 cases during the winter of 2024 [3], obtaining clinical data reporting the clinical phenotype and lethality of patients infected with this subvariant as compared with the previous ones is crucial to inform public health authorities and clinicians managing these patients. Our data provide reassuring evidence regarding the severity of disease associated with JN.1 infection, showing not only a non-significant difference in day 28 mortality compared to patients infected with XBB, but also no significant differences in other outcomes, including the need for invasive mechanical ventilation and length of stay in the ICU. Consistently, there was also no significant association between sublineage and day-28 mortality in uni- and multivariable logistic regression analysis.

Patients infected with sublineage JN.1 were more likely to be obese and less likely to be immunosuppressed than those infected with XBB in our study. Such a finding was unexpected because immunosuppression has been reported to be the most common comorbidity in COVID-19 patients infected with the Omicron variant since the “ancestral” BA.1 Omicron sublineage [4, 13], occurring in almost 50% of cases, and may reflect an inherently less pathogenic variant as reported in a hamster model [14]. On the other hand, the higher prevalence of obesity, a previously reported risk factor for severity with previous SARS-CoV-2 variants, including the ancestral variant, is consistent with previous data reporting obesity as a risk factor for severity [15]. These findings may have important implications for the updated use of pre-exposure monoclonal antibodies use [16] as well as COVID-19 vaccination recommendations. Initial estimates of the updated XBB.1.5 COVID-19 vaccine showed sustained vaccine efficacy against symptomatic JN.1 lineage infection [17].

The day-28 mortality rate measured in the current cohort (JN.1 group: 14.6%; XBB group: 22.0%) of critically ill COVID-19 patients was numerically lower than that of previously published cohorts involving other variants (i.e., Wuhan: 31% [18]; Alpha: 26% [19]; Delta: 29% [4]) or Omicron sublineages (i.e., BA.1: 35%; BQ.1.1: 22% [4, 5]), suggesting a milder severity of JN.1 infection in the ICU. Such finding is corroborated by the fact that only one third of JN.1-infected patients required invasive mechanical ventilation and extra-corporeal membrane oxygenation support was almost never required, despite the fact that the majority of patients were categorized as having SARS-CoV-2 pneumonia at cause for acute respiratory failure (as opposed to SARS-CoV-2-associated cardiogenic pulmonary edema or decompensated chronic respiratory failure). The higher prevalence of obesity in JN.1- than in XBB-infected patients might in part account for the numerically better outcome in the former patients, as previously demonstrated [20]. In contrast, patients with a lower body mass index had a higher risk of day-28 mortality. In terms of ICU management, patients in the JN.1 group received dexamethasone more frequently than their counterparts in the XBB group, possibly because they were less likely to be immunosuppressed. Other aspects of treatment did not differ.

Our study certainly has limitations, including a limited sample size in the JN.1 group, which limits our statistical power to perform subgroup analyses and adjust for confounding variables. The generalizability of our findings is also restricted to the population studied. Indeed, we included two groups of critically ill patients who showed no statistically significant mortality difference. We thus cannot exclude any populational difference in disease severity, with associated risks of hospitalization, ICU admission and death, related to the infecting Omicron sublineage. Combined with the observational nature of our study design, we also did not define a priori the sample size of the study, making our findings exploratory. This is because SEVARVIR aims at capturing the dynamics of emerging SARS-CoV-2 sublineages and analyzing their phenotype and relationship with mortality in real time. However, our study also has major strengths, in particular the constitution of a unique national prospective multicenter cohort of well-phenotyped critically ill patients and the availability of full-length SARS-CoV-2 genome sequences, allowing for prospective exploration of the clinical consequences of emerging and spreading SARS-CoV-2 sublineages.

In conclusion, our exploratory analysis of critically-ill patients with Omicron JN.1 infection suggested a different clinical phenotype than patients infected with the earlier XBB sublineage, including more frequent obesity and less immunosuppression. Compared with XBB, JN.1 infection was not associated with higher day-28 mortality.

### Supplementary Information

Supplementary Material 1.

## Data Availability

Raw data are available on reasonable request to the corresponding author.

## Abbreviations

ARDS: Acute respiratory distress syndrome
COVID-19: Coronavirus disease 2019
ICU: Intensive care unit
RT-PCR: Reverse transcriptase-polymerase chain reaction
SARS-CoV-2: Severe acute respiratory syndrome coronavirus 2
SAPS II: Simplified acute physiology score II score
SOFA: Sequential organ failure assessment
WHO: World Health Organization
